# 40 Hz audiovisual stimulation improves sustained attention and related brain oscillations

**DOI:** 10.1162/IMAG.a.1229

**Published:** 2026-05-13

**Authors:** Matthew K. Attokaren, Lu Zhang, Sindhura Mettupalli, Annabelle C. Singer

**Affiliations:** Coulter Department of Biomedical Engineering, Georgia Institute of Technology & Emory University, Atlanta, GA, United States; National Institute of Mental Health, National Institutes of Health, Bethesda, MD, United States

**Keywords:** gamma sensory stimulation, vigilance, attention, delta oscillations, alpha oscillations, functional connectivity

## Abstract

Gamma oscillations (30–100 Hz) have long been theorized to play a key role in sensory processing and attention by coordinating neural firing across distributed neurons. Gamma oscillations can be generated internally by neural circuits during attention or exogenously by stimuli that turn on and off at gamma frequencies. However, it remains unknown if driving gamma activity via exogenous sensory stimulation affects attention. We tested the hypothesis that non-invasive audiovisual stimulation in the form of flashing lights and sounds (flicker) at 40 Hz improves attention in an attentional vigilance task and affects neural oscillations associated with attention. We recorded scalp EEG activity of healthy adults (n = 62) during 1 hour of either 40 Hz audiovisual flicker, no flicker as control, or randomized flicker as sham stimulation, while subjects performed a psychomotor vigilance task. Participants exposed to 40 Hz flicker stimulation had better accuracy and faster reaction times than participants in the control groups. The 40 Hz group showed increased 40 Hz activity compared to the control groups in agreement with previous studies. Surprisingly, 40 Hz subjects had significantly lower delta power (2–4 Hz), which is associated with arousal, and higher functional connectivity in lower alpha (8–10 Hz), which is associated with attention processes. Furthermore, decreased delta power and increased lower alpha functional connectivity were correlated with better attention task performance. This study reveals how 40 Hz audiovisual stimulation improves attention performance with potential implications for therapeutic interventions for attention disorders and attention improvement.

## 1 Introduction

Sensory stimulation in the gamma band, lights and sounds that turn on and off at 40 Hz, or “40 Hz flicker”, has recently gained interest as a potential non-invasive intervention for Alzheimer’s disease (Bartolini et al., 2025; Chan et al., 2022; Hansen et al., 2024; He et al., 2021; Iaccarino et al., 2016; Martorell et al., 2019; Murdock et al., 2024). Multiple studies have shown that chronic 40 Hz flicker (also called gamma sensory stimulation) over days or weeks reduces Alzheimer’s pathology, including amyloid beta, alters neuroimmune activity, such as microglia function and cytokine signaling, and enhances glymphatic clearance in mice (Iaccarino et al., 2016; Martorell et al., 2019; Murdock et al., 2024; Singer et al., 2018; Zhou et al., 2024). Recent and ongoing studies are determining whether 40 Hz flicker has beneficial effects in human patients with Alzheimer’s disease, epilepsy, and other diseases (Bartolini et al., 2025; Blanpain et al., 2024; Chan et al., 2022; He et al., 2021). While prior studies have focused on the effects of chronic 40 Hz flicker in disease, little is known about 40 Hz flicker’s acute influences on cognition in healthy adults. A key function of endogenous gamma oscillations is to facilitate attention processing (Bauer et al., 2006; Bosman et al., 2012; Fries et al., 2001; Gregoriou et al., 2009; Herrmann, 2001; Tiitinen et al., 1993; Womelsdorf et al., 2006), but how acute 40 Hz flicker might affect attention is unclear. Indeed, whether sensory-induced gamma enhances, coexists with, or disrupts ongoing oscillations and related cognitive functions is a topic of debate (Duecker et al., 2021; Soula et al., 2023).

Gamma oscillations (which can include oscillatory activity from 30–100 Hz) have long been theorized to play a key role in sensory processing and attention by coordinating firing across distributed neurons (Buzsáki & Wang, 2012). Attention is thought to be facilitated by internally generated gamma oscillations because this activity coordinates neurons across brain regions to fire together on short timescales, thus enhancing communication between regions (Bosman et al., 2012; Clayton et al., 2015; Jensen et al., 2007; Tallon-Baudry et al., 1998). Specifically, neurons that respond to the attended stimulus exhibit increased synchronization in the gamma frequency range compared to other neurons, and this synchronized activity is more likely to drive downstream activity (Fries et al., 2001). While gamma oscillations observed during attention are internally generated by neural circuits, gamma frequency activity is also elicited by exogenous stimuli that turn on and off at gamma frequencies, like 40 Hz (Attokaren et al., 2023; Herrmann, 2001). Current studies have debated whether exogenously driven gamma oscillations from rhythmic sensory stimuli have roles overlapping with those of gamma oscillations generated internally. Some theories posit that entrainment of neural activity to sensory inputs enhances representations of attended stimuli (Obleser & Kayser, 2019). A subset of evidence suggests that rhythmic sensory inputs entrain endogenous oscillations, while other evidence indicates that sensory-driven and endogenous oscillations separately co-exist (Duecker et al., 2021, 2024; Obleser & Kayser, 2019; van Bree et al., 2021; Zoefel et al., 2018). Furthermore, flashing lights and sounds like those used during flicker may enhance sensory entrainment or distract an individual from a cognitive task. Thus, it is unclear if 40 Hz flicker stimulation would enhance sensory entrainment and attentional processing or if such stimulation would disrupt endogenous oscillations and distract an individual.

In this study, we tested the hypothesis that non-invasive sensory stimulation in the form of flashing lights and sounds at 40 Hz improves attentional vigilance and modulates neural oscillations and functional connectivity associated with attention processing. We exposed 62 healthy young adults to 40 Hz audiovisual flicker or control conditions while they performed a vigilance task, and we recorded EEG activity to assess neural dynamics during stimulation and attention processing. One control group received sham stimulation: a random, non-periodic flicker stimulation that had the same average on-time as the 40 Hz group to control for the effects of a flashing stimulus. The other control group was exposed to constant light that was measured to be the same brightness as the 40 Hz flicker stimulation. We compared the 40 Hz group’s vigilance task performance, including reaction times and accuracy, to the control groups. We assessed brain oscillations, including power and functional connectivity, and oscillations both in and outside of the gamma band, while subjects attended to the stimulus during the task. Finally, we determined which changes in oscillations correlated with improved task performance. Together, these findings elucidate how 40 Hz flicker affects attentional processing.

## 2 Methods

### 2.1 Participants

The study was approved by the Institutional Review Board at the Georgia Institute of Technology. Participants (32 males, 30 females) between the ages of 18 and 40 were analyzed in this study (Supplementary Table S1). Participants were screened for eligibility based on the inclusion criteria of right-handedness. Exclusion criteria were conditions or medications that would affect blood flow and/or neural processes, color blindness and/or uncorrected vision, unremovable metal in the form of things like piercings or implants of any kind, history of neurological or psychological disorders/diseases, and any other conditions that could potentially affect the EEG results. Participants were also screened for any conditions that may necessitate exclusion from our EEG study for safety or technical reasons including being unable to sit for long periods of time or being unable to tolerate flashing lights. One hundred eighty-four people submitted paperwork required for us to determine if they met inclusion criteria. Of those, 52 were excluded for not meeting inclusion criteria (described above), and 56 were excluded for not meeting the scheduling needs of the experiment. Of those that participated in the experiment, 6 were excluded for technical problems during EEG recording. Additional subjects were excluded following data analysis for outlier behavioral performance (8 subjects).

All participants provided written and verbal consent prior to their session and were informed of any potential risks that could occur as a result of study procedures. Participants were compensated monetarily or with class credit for their participation in the study. Participants were randomly assigned to one of the three stimulation groups: 40 Hz, Random, and Constant Light (also called “Light”). We found no significant differences in the sample demographics between groups (Supplementary Table S1). Prior to the study, participants were asked to ensure they kept to their normal routines to minimize changes in their daily practices that could influence their EEG session. Participants were surveyed immediately before and after their session to assess changes in drowsiness, dizziness, and boredom during the study.

### 2.2 Flicker exposure

**Fig. 1.**
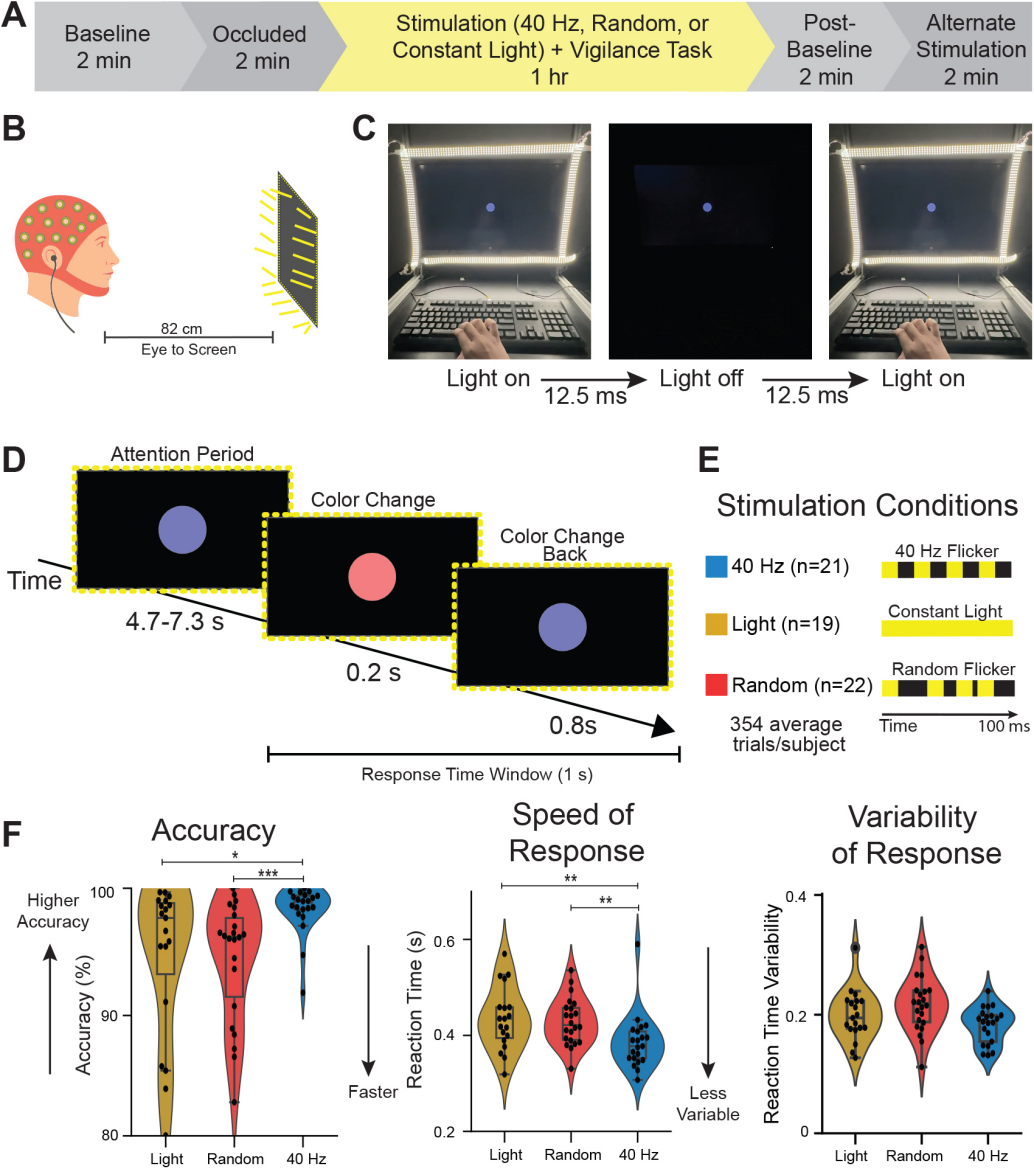
40 Hz flicker improved accuracy and reaction time in a vigilance task. (A) Experimental timeline. EEG data were recorded from participants in a baseline period, occluded stimulation period, and during sensory exposure and task performance. (B) Illustration of a person undergoing audio and visual stimulation while performing the computer-based psychomotor vigilance task. (C) For the 40 Hz flicker group, LEDs on the frame of the monitor turn on and off every 12.5 msec during the task. (D) In the task, subjects looked at a blue dot on the screen (“Attention Period”). At an unpredictable time, the dot changed to red for 0.2 seconds (“Color Change”). After the 0.2 seconds ended, the color changed back to blue (“Color Change Back”). If the subject detected the color change, they pressed a key on a keyboard as quickly as possible and within 1 second of the color change (“Response Time Window”). This schematic shows 1 trial. Subjects performed on average 354 ± 6.76 trials over 1 hour. (E) Subjects either underwent 40 Hz flicker (n = 21 subjects, blue) in which the LED frame light turned on and off at 40 Hz (on for 12.5 msec and off for 12.5 msec), a control of constant light (n = 19 subjects, gold) in which the light stayed on throughout the test, or random flicker (n = 22 subjects, red) as a sham condition in which the light turned on for 12.5 msec but turned off for randomized intervals. The random sham stimulation controls for effects of a flickering stimulus, like salience. (F) *Left:* Distribution of accuracy for each participant per stimulation group showed that 40 Hz flicker (blue) improved accuracy (Kruskal-Wallis test: χ²(2) = 7.98, p = 0.018) in the vigilance task compared to both Light (gold, control; p = 0.018, post-hoc ranksum test; q < 0.05, FDR correction for 2 comparisons) and Random flicker (red, sham; p = 0.00056, ranksum test; q < 0.05, FDR correction for 2 comparisons) with a significant effect of group. The violin plot shows the histogram of the full distribution with the central box plot spanning the 25^th^ percentile to the 75^th^ percentile of the data. The horizontal line inside the box indicates the median, and the whiskers extend to the smallest and largest values within 1.5 times the interquartile range. Each black dot is one subject. *Center:* Distribution of reaction times for each stimulation group shows 40 Hz flicker reduced reaction time (Kruskal-Wallis test: χ²(2) = 7.06, p = 0.029) in the vigilance task compared to both Light (control; p = 0.0051, ranksum test; q < 0.05, FDR correction for 2 comparisons) and Random flicker (sham; p = 0.0028, ranksum test; q < 0.05, FDR correction for 2 comparisons) with a significant effect of group. Distribution of average reaction time variability did not have a significant effect of group (Kruskal-Wallis test: χ²(2) = 4.25, p = 0.120). Asterisks indicate: *p < 0.05, **p < 0.01, ***p < 0.001.

Participants were separated into three groups: 40 Hz (n = 21), Random (n = 22), and Constant Light (n = 19) Stimulation (Fig. 1A). To administer the different stimulation types, participants were seated in front of a computer monitor with a frame of light-emitting diodes (LEDs) around the border that turned on and off with millisecond precision (Fig. 1B). For the 40 Hz group, during the 1-hour stimulation period, participants were exposed to LEDs and sound delivered via ear buds that turned on and off every 12.5 msec (50% duty cycle) to generate 40 Hz stimulation (Fig. 1C). For the Random group, the LEDs and audio were set to turn on and off at a non-periodic, variable rate that maintained the same average number of on-off transitions as the 40 Hz group. For the Constant Light group, the LEDs remained continuously on without modulation, and no audio was provided. Participants did not know if they were in a control or treatment group. They were not informed about different stimulation groups, and all subjects were exposed to flickering stimuli at some point over the course of the experiment, thus they were unlikely to deduce their group assignment even though the stimulus was detectable. Due to the flicker stimulus being visible, experimenters were not blind to group during data collection. All subjects were analyzed together using the same processing pipeline and thus experimenters were blind during analysis.

### 2.3 Study design and EEG recording

EEGs were recorded using a 32-channel BioSemi Active Two system with data acquired at a sampling rate of 2048 Hz. In addition to the 32-channel electrode cap, 6 facial electrodes were used including two placed on the mastoids. During each recording, all room lights were turned off, except for the frame of LEDs during the 1-hour stimulation portion. The door was closed to shield the participant from light and noise from outside the room. Participants were instructed not to excessively blink, move around unnecessarily, or cross their legs during the study to minimize noise due to muscle movements. EEG data were synchronized to behavior data using TTL parallel port event triggers delivered to the EEG system from the PsychoPy software.

In the study, subjects first underwent a baseline recording followed by an “occluded” recording designed to detect electrical artifacts of stimulation (Fig. 1A). During the occluded recording, participants wore an eye mask and earplugs while flicker stimulation ran to ensure that detected changes in EEG power in recordings were a result of changes in brain activity rather than a byproduct of electrical noise from stimulation devices. During the 1-hour stimulation period, subjects performed a psychomotor vigilance task (PVT, described below).

Only subjects showing that 40 Hz audiovisual flicker increased 40 Hz neural activity in some EEG channels were included in further analysis, in line with previous studies in humans (Attokaren et al., 2023; Herrmann, 2001; Pastor et al., 2002). To assess whether subjects exhibited such increases in 40 Hz activity in response to 40 Hz flicker, participants who were not in the 40 Hz group were briefly shown 40 Hz stimulation after the end of their 1-hour control or sham stimulation EEG session. For each EEG channel, 40 Hz activity was considered significantly increased in response to flicker if power at 40 Hz exceeded the average power in nearby frequencies (31–39 and 41–49 Hz) by at least three standard deviations. For an individual subject, significant modulation by 40 Hz was defined by the presence of at least three modulated channels, with at least one modulated channel in each hemisphere (Attokaren et al., 2023). Participants who did not exhibit significant 40 Hz modulation were excluded from analysis (n = 2).

### 2.4 Psychomotor vigilance task (PVT)

To assess sustained attention (i.e., vigilance) during stimulation, participants completed a PVT presented via PsychoPy. Participants were instructed to focus on a dot at the center of the screen and respond whenever the dot’s color changed from blue to red (Fig. 1D). The “attending period”, or the period when the dot remained blue before the color change, lasted for 4.7–7.3 seconds with the duration jittered per trial to be unpredictable. Then, the dot changed colors from blue to red for 0.2 seconds, before switching back to blue for 0.8 seconds, after which a new trial began. During the 1 hour of the PVT and EEG recording, participants were given short breaks from the task approximately every 20 minutes, during which they remained at the monitor and continued receiving sensory exposure (Fig. 1E).

### 2.5 Behavior analyses

Each PVT trial was categorized as: (1) a premature response if the participant responded before the color change occurred; (2) a hit if the participant responded within 1 second after the color change occurred; or (3) a miss if the participant did not respond within that 1-second window. Accuracy was computed as the number of hit trials out of total trials and reaction time as the time from color change to a button press. No reaction time was recorded for missed trials or premature responses. Participants with an accuracy below 80% were considered outliers (8 in total) and excluded from behavior analyses. This ensured that only participants who were actively engaged in the task and thus continuously receiving stimulation were included in the behavior analysis.

### 2.6 EEG data analyses

Prior to analysis, EEG data were preprocessed to remove noise artifacts such as eye blinks and muscle movements, identified using independent component analysis (ICA), and faulty channels using the EEGLAB toolbox for MATLAB. A high-pass filter at 1 Hz was applied. Data were segmented into epochs, one epoch per trial. Each epoch spanned from 4 to 0 seconds prior to the color change.

To measure the distribution of power contained within the EEG signal over frequency for a given EEG channel, power spectra were calculated using Welch’s method (Welch, 1967), with 50% overlapping Hamming windows of 2 seconds length and Fast Fourier Transform (FFT) size of 1024, implemented with MATLAB’s Signal Processing Toolbox. We normalized the raw power to reduce the effects of individual variation in power. This normalization enables the assessment of relative increases or decreases in power from a similar baseline. To normalize power, we divided raw power by the area under the power spectral density from 1–55 or 2–55 Hz. We used two different ranges (1–55 Hz or 2–55 Hz) because we noticed differences between groups near 1 Hz and wondered if those differences might be driving differences between groups. Normalizing using the area under the power spectral density from 2–55 Hz excluded potential effects at 1 Hz. Importantly, we found similar results when normalizing the power spectral density from 1–55 Hz and measuring delta from 1–4 Hz and when normalizing the power spectral density from 2–55 Hz and measuring delta from 2–4 Hz, indicating that differences around 1 Hz were not driving the overall effects observed.

For visualization of EEG power on scalp maps, FOOOF (fitting oscillations & one over f) was applied to remove aperiodic spectra (https://fooof-tools.github.io/fooof/). FOOOF uses a model-driven approach to separate and quantify periodic (oscillatory) and aperiodic (1/f-like) activity (Donoghue et al., 2020). Using this approach, we removed aperiodic components. We took this approach because a difference in aperiodic components could create seeming differences in periodic activity (Donoghue et al., 2020).

We measured functional connectivity using weighted phase lag index (WPLI) to minimize the impact of volume conduction of source activity (Vinck et al., 2011). The WPLI varies between 0 and 1 with 0 indicating either no phase coupling or a balanced distribution of leading and lagging phases while 1 denotes the phase of one signal consistently leading the other. WPLI quantifies the imaginary component of coherence using the following formula (Vinck et al., 2011):

$$WPLI_{xy}\left(f\right)=\frac{\left|E\left\{Im\left(S_{xy}\left(f\right)\right)\right\}\right|}{E\left\{\left|Im\left(S_{xy}\left(f\right)\right)\right|\right\}}$$,

where $S_{xy}\left(f\right)$ is the cross-spectrum between signals recorded from two EEG channels, x(t) and y(t) as a function of frequency f, where E{⋅} represent cross-trial average, Im(⋅) is the Imaginary component. WPLI was computed for every pair of channels within each subject for all attention period epochs.

To identify EEG channel pairs with significant functional connectivity, we compared connectivity values to a null distribution created using a permutation-based approach. In each permutation, we randomly selected an EEG channel from each of two subjects (not necessarily the same channel in both subjects), then computed the WPLI for the resulting channel pair. This process was repeated 10,000 times to generate a null distribution of connectivity values, as genuine connectivity should not exist across subjects. Real WPLI values were then compared to this null distribution, with WPLI values exceeding the 99.99^th^ percentile (p < 0.0001) considered significant. We quantified the total number of significant channel pairs for each frequency band and for each group to identify which bands were significantly modulated by 40 Hz flicker exposure.

### 2.7 Statistical approach

In general, a false discovery rate of 0.05 was applied for multiple comparisons. Group comparisons of behavioral measures were assessed using the Kruskal-Wallis test as a one-way non-parametric ANOVA. Ranksum tests with FDR correction were used for post-hoc analysis for two comparisons (40 Hz versus Random and 40 Hz versus Light). Our hypothesis was that 40 Hz stimulated subjects would differ from those that were exposed to Random and Light, and therefore we restricted our analyses to those hypothesis-driven comparisons, consistent with best practices (Maxwell et al., 2017; Tukey, 1991). We used a Kruskal-Wallis test for behavioral analyses instead of an ANOVA because the distributions were not normally distributed. Group comparisons of EEG power were conducted using two-way and three-way mixed ANOVAs, with stimulation group as a between-subjects factor and frequency band and channel as within-subjects (repeated-measures) factors. Post-hoc tests to compare EEG power in delta, theta, alpha, beta, and gamma were unpaired, two-sided t-tests corrected for 30 comparisons (2 group comparisons: 40 Hz versus Random and 40 Hz versus Light, 3 channels, and 5 frequency bands). Comparisons in the Gamma Flicker Response band (39–41 Hz) were assessed separately because that band reflects strong sensory drive.

## 3 Results

### 3.1 40 Hz flicker improved accuracy and reaction time in a vigilance task

Internally generated gamma oscillations are thought to enhance circuit computations for attention (Bauer et al., 2006; Börgers et al., 2005; Herrmann & Mecklinger, 2001; Jensen et al., 2007); however, the effects of sensory-induced gamma on attention are unknown. Accordingly, we asked how 40 Hz flicker affects accuracy and reaction time in an attentional vigilance task in which subjects must attend to a stimulus over time to detect and respond to an unpredictable color change. To address this question, we exposed subjects to 40 Hz sensory flicker stimulation (40 Hz), constant light (Light) as a control, or random flicker stimulation (Random) as a sham stimulation during a psychomotor vigilance task (PVT) in which participants watching a dot on a screen were instructed to press a key in response to the dot changing colors. Participants with accuracy below 80% were considered not engaged in this simple task and were excluded from the behavior analysis. Constant light served as a control to see how 40 Hz affects vigilance compared to a steady lighting environment, and random flicker was used as sham stimulation to control for the effects that flashing lights and sounds may cause irrespective of their flickering frequency. We tested the hypothesis that 40 Hz stimulation altered behavior compared to Random and Light, and we restricted our analyses to those comparisons. Subjects in the 40 Hz group had a significantly higher accuracy than those in the Light and Random groups during the PVT (Fig. 1F, left; Kruskal-Wallis test: χ²(2) = 7.98, p = 0.018; post-hoc ranksum test: 40 vs. Light: p = 0.018; 40 vs. Random: p = 0.00056; q < 0.05 FDR correction for 2 comparisons). Mean accuracy was 98.4%, 94.7%, and 94.5% for 40 Hz, Light, and Random groups, respectively. Surprisingly, the 40 Hz group also reacted more quickly than the other two groups (Fig. 1F, middle; Kruskal-Wallis test: χ²(2) = 7.06, p = 0.029; post-hoc rank sum tests: 40 vs Light: p = 0.0051; 40 vs. Random: p = 0.0028; q < 0.05, FDR correction for 2 comparisons). Reaction times during 40 Hz flicker were on average 11.3% faster than during constant light and 10.0% faster than during Random. Importantly, a speed-accuracy tradeoff (Heitz, 2014), or a higher accuracy correlated with slower reaction time, was not observed (Supplementary Fig. S1). We found no significant effect of stimulation group on reaction time variability (Fig. 1F, right; Kruskal-Wallis test: χ²(2) = 4.25, p = 0.120). These findings show that 40 Hz flicker enhances vigilance as indicated by higher accuracy and faster reaction time compared to constant light or sham stimulation.

### 3.2 40 Hz flicker is correlated with decreases in delta activity during a vigilance task

**Fig. 2.**
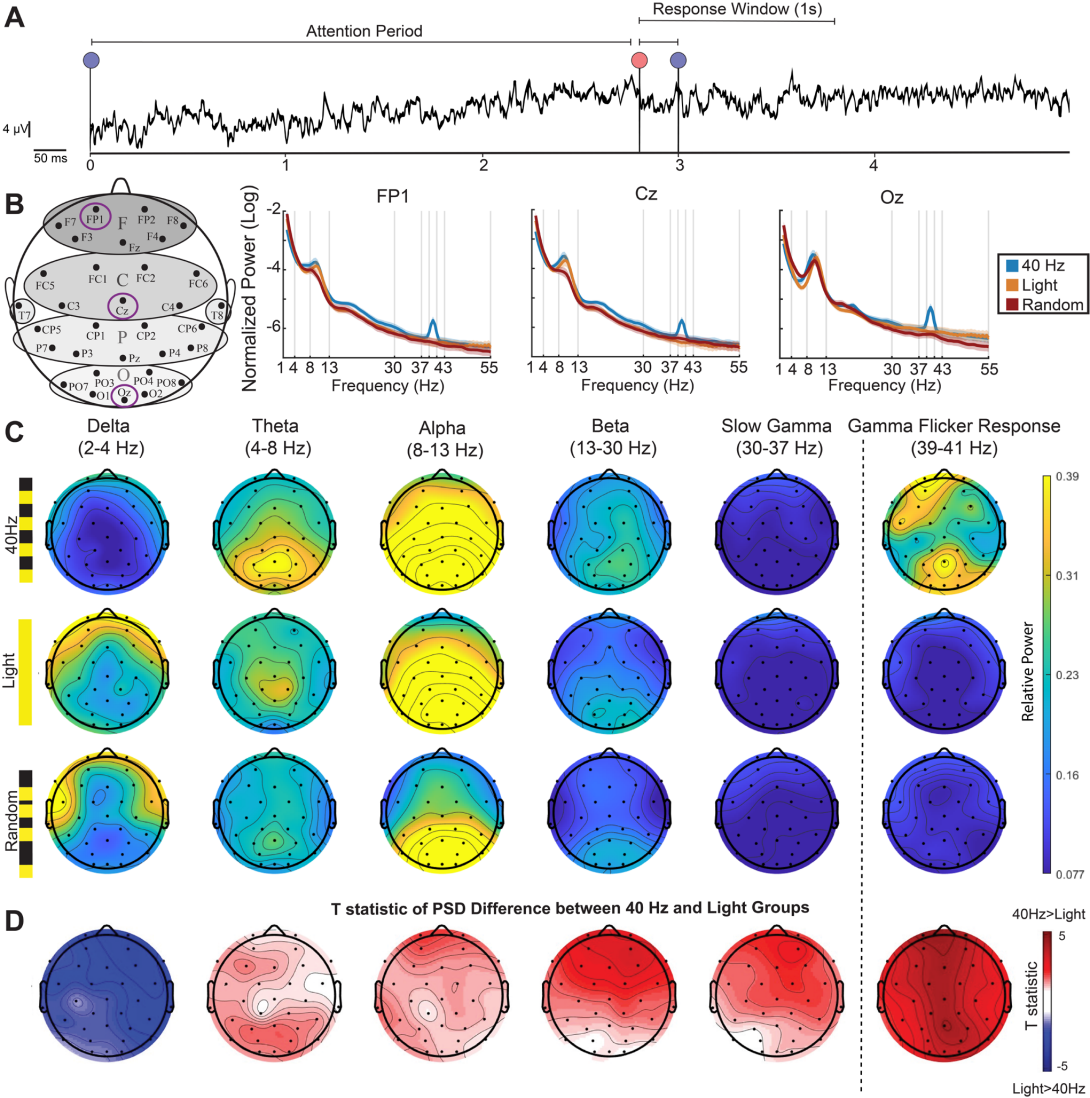
40 Hz flicker decreased delta activity during a vigilance task. (A) EEG trace from a single channel during a single trial including the attention period, followed by the color change period, and concluding with the response window. (B) *Left:* Topographic map showing the locations of three selected EEG channels (FP1, Cz, Oz). We selected these three channels (Fp1, Cz, and Oz) to capture EEG activity associated with prefrontal/frontal cognitive activity, central sensorimotor processing, and occipital sensory responses. *Right:* corresponding power spectral density (PSD) plots in the 40 Hz (blue), constant light (orange), and Random (red) groups. Each PSD shows mean ± SEM. (C) Topographic maps of periodic components of PSDs extracted using FOOOF (see Section 2) for each stimulation group—40 Hz (top row), Light (middle row), and Random (bottom row)—across EEG frequency bands of interest: delta (2–4 Hz), theta (4–8 Hz), alpha (8–13 Hz), slow gamma (30–37 Hz), and the Gamma Flicker Response band (39–41 Hz). Warmer colors indicate higher power. (D) *T*-statistics of the difference between the real PSD of the 40 Hz group and the Light group during the attention period. Red indicates power in the 40 Hz group is higher than the Light group (positive *t*-statistics), while blue indicates power in the 40 Hz group is lower than the Light group (negative *t*-statistics) (two-sided *t*-test).

Because participants undergoing 40 Hz flicker performed better on a vigilance task than control and sham stimulation groups, we wondered how 40 Hz flicker affected multiple oscillation bands that are implicated in attention processing. As described above, endogenous gamma oscillations increase during attention tasks (Bauer et al., 2006; Jensen et al., 2007). In contrast, elevated delta oscillations (1–4 Hz) have been linked to reduced vigilance, cognitive disengagement, fatigue, attention deficits, increased cognitive workload, decreased alertness, and transitions toward sleep-like states (Chang et al., 2013; Ogilvie, 2001; Ogilvie et al., 1991; Oken et al., 2006). Higher power in beta oscillations (13–30 Hz) is thought to play a role in motor control, sustained attention, and visual attention (Engel & Fries, 2010; Wróbel, 2000), while higher power in alpha oscillations (8–13 Hz) has been linked to inhibition including the suppression of irrelevant sensory input (Jensen & Mazaheri, 2010; Klimesch, 2012). To determine how these oscillations associated with attention differ between 40 Hz flicker and control groups, we recorded neural activity using a 32-channel scalp EEG on participants in the 40 Hz, Light, and Random groups while they performed the vigilance task during stimulation as described above. To identify neural oscillations associated with attention, we examined neural activity during the attention period preceding the color change of the dot on Hit trials (Fig. 2A). To perform an unbiased analysis across multiple frequency bands, we measured peak power within five frequency bands: delta (2–4 Hz), theta (4–8 Hz), alpha (8–13 Hz), beta (13–30 Hz), and low gamma (30–37 Hz) as well as around the flicker frequency (39–41 Hz, or “Gamma Flicker Response” band). We noticed that the averaged raw power spectral density in the 40 Hz group shows a different baseline or 1/f than the random or Light groups, indicated by elevated power across frequencies from about 4–50 Hz (Supplementary Fig. S2B). Because this increase appears to be a baseline offset rather than a specific increase in power of periodic activity, we could not measure differences between groups in raw power alone as that would conflate differences due to periodic and aperiodic components of the activity (Donoghue et al., 2020). As a result, we used two different approaches to assess changes in periodic activity: normalizing power spectral density to focus on relative increases or decreases in power from a similar baseline and a Fitting Oscillations & One-Over-F (FOOOF) visualization which separates periodic from aperiodic components (see Section 2). We measured normalized power to control for individual differences in overall power. We normalized from 2–55 Hz to minimize the effects of differences in low frequencies (1–2 Hz) on the normalization (see raw power spectral densities in Supplementary Fig. S2B). We repeated analyses normalized from 1–55 Hz and with delta defined as 1–4 Hz and found similar results. The results shown used power normalized from 2–55 Hz, with delta defined as 2–4 Hz. We separately measured power around 40 Hz (39–41 Hz, Gamma Flicker Response) as rhythmic sensory stimuli are known to induce a steady state evoked potential (Herrmann, 2001). Consistent with prior studies showing periodic flicker increases power in neural activity at the flicker frequency, we found that participants in the 40 Hz group had elevated power in the Gamma Flicker Response band (Fig. 2B–D; Supplementary Fig. S2A–E; two-way mixed ANOVA: F_6, 189_(Channel × Group) = 31.50, p < 0.001; 40 Hz vs. Light: Fp1 p = 0.00017, Cz p = 0.00048, Oz p = 0.00095, T8 p = 0.059; 40 Hz vs. Random: Fp1 p = 7.50 × 10^−5^, Cz p = 0.0033, Oz p = 7.06 × 10^−5^, T8 p = 0.050; unpaired two-sided *t*-test not corrected for multiple comparisons). Indeed, visualizing the periodic components of EEG power on scalp maps, using FOOOF, we observed that the Gamma Flicker Response band (39–41 Hz) relative power was greater than 0.2 on all channels but one during 40 Hz flicker, while it was less than 0.1 on most channels during Random or Light conditions (Fig. 2C, far right).

We investigated whether there were differences in EEG activity outside the steady state evoked potential expected at 40 Hz. To visualize power across frequency bands in different groups, we used the FOOOF approach to distinguish rhythmic components of the power spectra from concurrent aperiodic components (Donoghue et al., 2020) (Fig. 2C). To assess frontal, central, and posterior EEG activity during vigilance and stimulation, we compared power at Fp1, Cz, and Oz channels between 40 Hz flicker and control groups. We selected these three channels (Fp1, Cz, and Oz) to capture EEG activity associated with prefrontal/frontal cognitive activity, central sensorimotor processing, and occipital sensory responses. We were surprised to find lower power within the delta band during 40 Hz flicker compared to the control and sham groups (Fig. 2B–D; Supplementary Fig. S2A–E; two-way mixed ANOVA: F_4, 126_(Channel × Group) = 47.25, p < 0.001; post-hoc *t*-tests for 40 Hz vs. Light: Fp1 p = 0.0064, Cz p = 0.016016; 40 Hz vs. Random: Fp1 p = 0.0053, Cz p = 0.001, Oz p = 0.0055; unpaired *t*-test; q < 0.05, FDR correction from 30 comparisons from 2 group comparisons, 3 channels, and 5 frequency bands). We did not find consistent significant differences in other frequencies when correcting for multiple comparisons and comparing to both control groups, however we noticed trends of elevated alpha and beta power in the 40 Hz group (Fig. 2B–D; Supplementary Fig. S2A–E; three-way mixed ANOVA: F_2, 63_(Fre.bands × Group) = 9.26, p < 0.001; beta band 40 Hz vs Light: Fp1 p = 0.020; alpha band 40 Hz vs Random Cz: p = 0.016, unpaired two-sided *t*-test; q < 0.05, FDR correction from 30 comparisons from 2 group comparisons, 3 channels, and 5 frequency bands). These results show that 40 Hz sensory flicker not only increases power around the flicker frequency but also decreases delta power during attention.

### 3.3 Reduced delta band power is correlated with better behavior performance

**Fig. 3.**
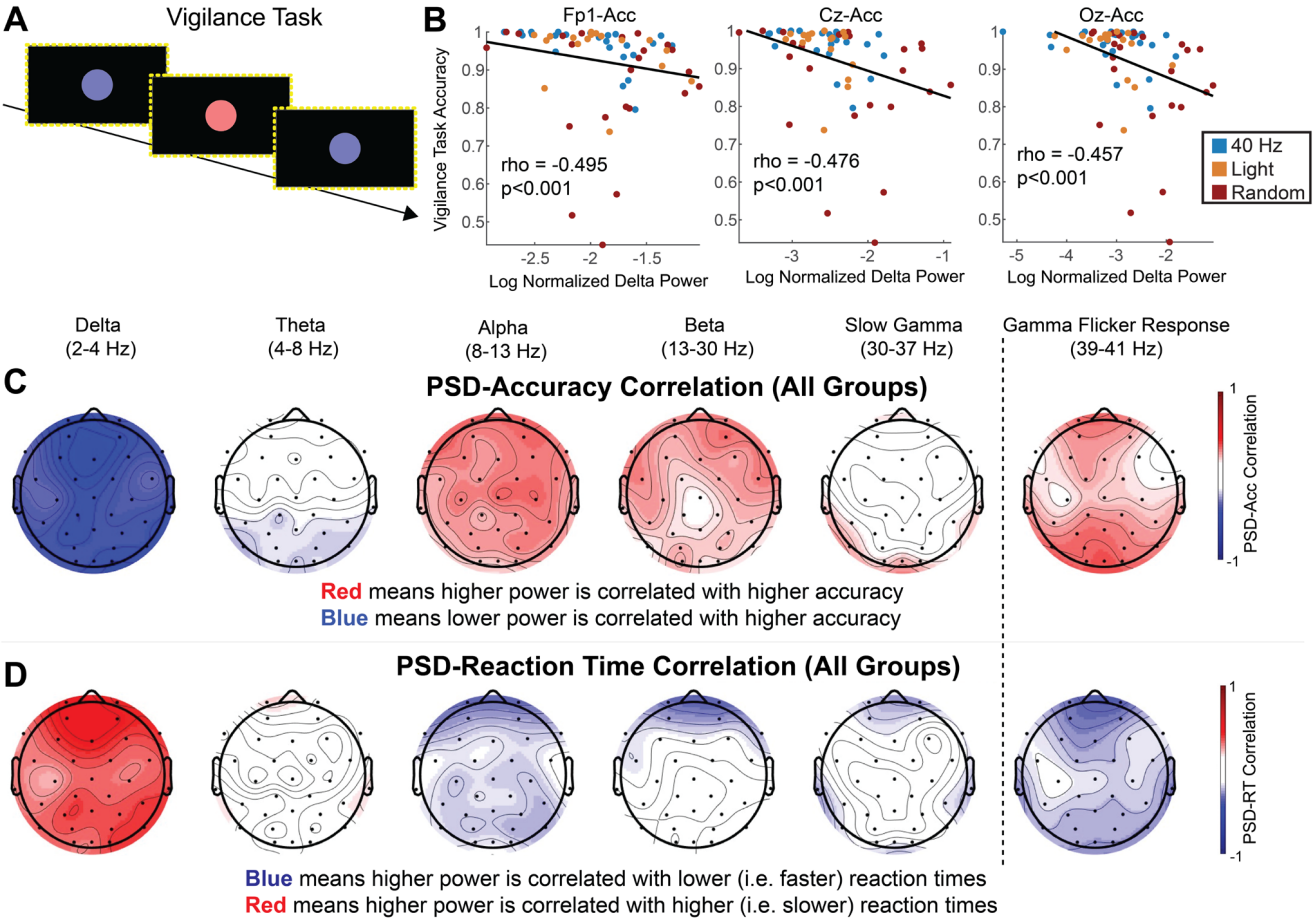
Reduced delta power is correlated with better accuracy and faster reaction time on a psychomotor vigilance task (PVT). (A) The sequence of images displayed during the PVT on a monitor with a border of LED lights. (B) Spearman’s rank correlation between delta power and accuracy correlation across all subjects on Fp1 (left), Cz (center), and Oz (right). Each dot represents one participant, with dot color indicating stimulation group (blue for 40 Hz, gold for Light, and red for Random). (C) Power-accuracy correlation topological maps including all experimental groups in the correlation for delta, theta, alpha, beta, slow gamma, and Gamma Flicker Response. Heatmap color shows Spearman’s rank correlation (rho) with red indicating higher power is positively correlated with higher accuracy and blue indicating a negative correlation. (D) As in (C) for power-reaction time correlation. Heatmap color shows Spearman’s rank correlation with blue indicating a negative power-reaction time correlation, that is, higher power occurs with faster reaction times or better performance, and red indicating a positive power-reaction time correlation, that is, higher power occurs with slower reaction times or worse performance.

We then asked how power in these different frequency bands correlated with performance on the psychomotor vigilance task (Fig. 3A). We determined if power and behavioral performance were correlated at three key EEG channels of interest: Fp1, Cz, and Oz (Fig. 3B). Higher accuracy was significantly correlated with lower delta band power and higher alpha band power (Fig. 3C; Supplementary Fig. S3A; delta band: Fp1 rho = -0.50, p = 3.2 × 10^−5^, Cz rho = -0.48, p = 7.1 × 10^−5^, Oz rho = -0.46, p = 0.00015; alpha band: Fp1 rho = 0.3517, p = 0.0044, Cz rho = 0.3428, p = 0.0056; Spearman’s rank correlation; q < 0.05, FDR correction for 15 comparisons, Supplementary Table S2). Reaction times were positively correlated with increased power in the delta band, meaning lower delta power occurred with faster reaction times. (Fig. 3D; Supplementary Fig. S3B; Delta band: Fp1 rho = 0.4895, p = 5.1 × 10^−5^, Cz rho = 0.3359, p = 0.0069, Oz rho = 0.3228, p = 0.0096; Spearman’s rank correlation; q < 0.05, FDR correction from 15 comparisons, Supplementary Table S2). Reaction times were negatively correlated with increased power within alpha and beta bands meaning higher power in those bands occurred with faster reaction times (Fig. 3D; Supplementary Fig. S3B; Alpha band: Fp1 rho = -0.3337, p = 0.036; Beta band: Fp1 rho = -0.3136, p = 0.012; Spearman’s rank correlation; q < 0.05, FDR correction from 15 comparisons, Supplementary Table S2). In line with elevated power in the Gamma Flicker Response band in the 40 Hz group and those subjects performing better in the task, we found a positive correlation between Gamma Flicker Response band power and accuracy (Supplementary Fig. S3C; Gamma Flicker Response band: Fp1 rho = 0.349, p = 0.006, Cz rho = 0.249, p = 0.046, Oz rho = 0.394, p = 0.003; Spearman’s rank correlation; q < 0.05, FDR correction from 3 comparisons). We also found reaction times were negatively correlated with power in the Gamma Flicker Response band on Fp1 and Oz (Supplementary Fig. S3D; Gamma Flicker Response band: Fp1 rho = -0.317, p = 0.030, Cz rho = -0.180, p = 0.154, Oz rho = -0.273, p = 0.044; Spearman’s rank correlation; q < 0.05, FDR correction from 3 comparisons). These results show that decreases in delta activity induced by 40 Hz flicker correlated with better vigilance performance. Furthermore, increased alpha and beta activity, which were trended up during 40 Hz flicker, were also correlated with better performance. Finally, higher power in the Gamma Flicker Response band correlated with better performance in the vigilance task.

### 3.4 40 Hz flicker increases low-alpha functional connectivity which is correlated with better behavior performance

**Fig. 4.**
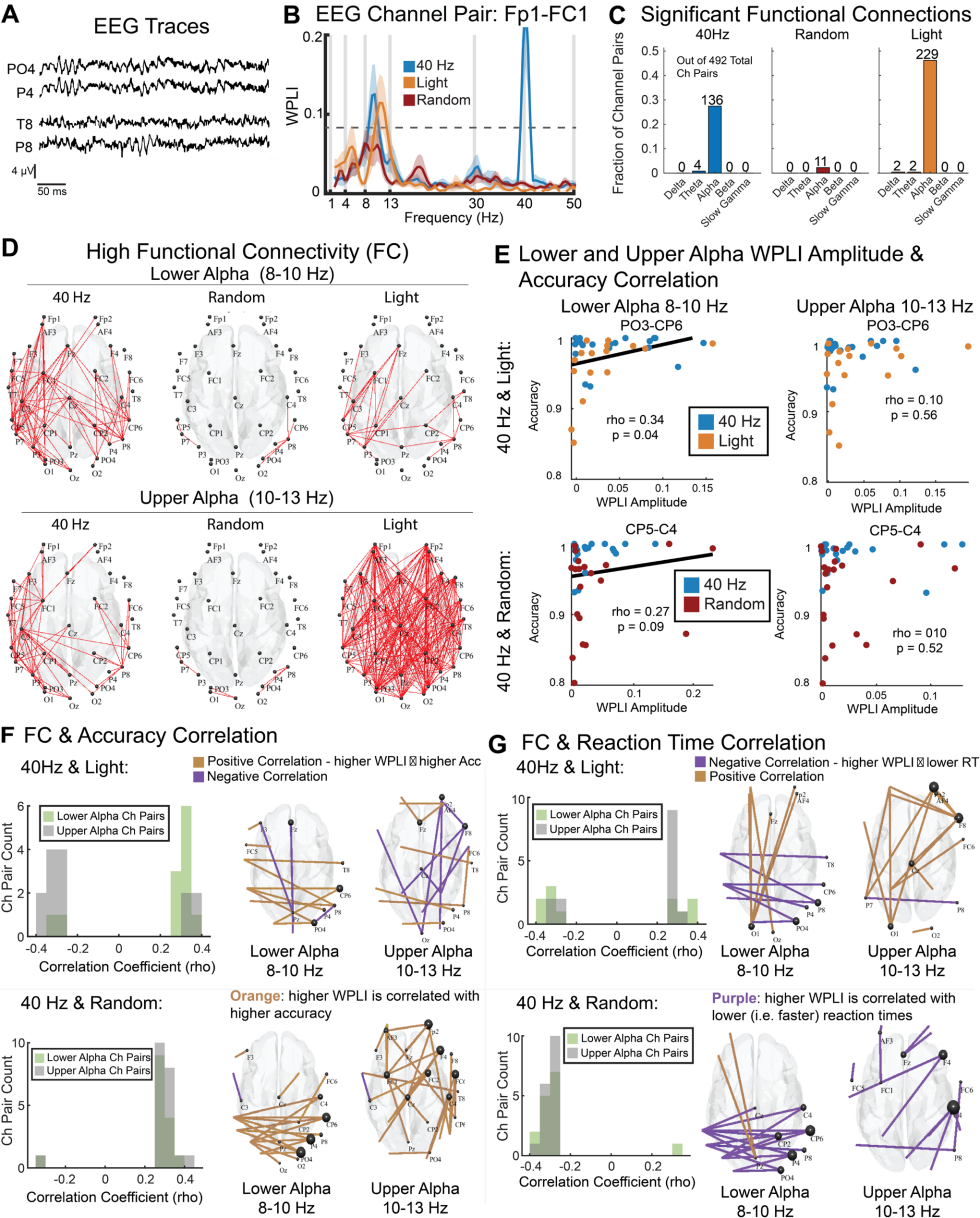
40 Hz flicker increased lower alpha functional connectivity, which correlated with better behavioral performance. (A) Representative EEG traces during attention period from two channel pairs with differing functional connectivity. The top panel shows two channels with high functional connectivity, while the bottom panel displays traces from two channels with low functional connectivity as measured via weighted phase lag index (WPLI). (B) WPLI as a function of frequency for an example channel pair, Fp1-FC1, across the three experimental groups: 40 Hz flicker (blue), Light (orange), and Random (red). (C) Number of EEG channel pairs with significant functional connectivity (p < 0.0001, WPLI > 0.089, permutation test) in each band of interest for each experimental group. (D) *Top:* EEG channel pairs (red lines) with high functional connectivity (WPLI values greater than the top quartile of WPLI channel pairs, i.e., WPLI > 0.1202) in the lower alpha band (8–10 Hz) in the 40 Hz (left), Random (center), and Light (right) groups. *Bottom:* As above for the upper alpha band (10–13 Hz). (E) *Top Left:* Scatterplot showing a representative EEG channel pair, PO3–CP6, in the lower alpha band (8–10 Hz) for 40 Hz and Light groups, with a positive correlation between WPLI amplitude and accuracy (rho = 0.34, p = 0.04, Spearman’s rank correlation), indicating that stronger functional connectivity is associated with higher accuracy. Each dot represents one subject, with orange for Light and blue for 40 Hz groups. *Top Right:* Scatterplot showing PO3–CP6 in the upper alpha band (10–13 Hz) for 40 Hz and Light groups, with no significant correlation (rho = 0.10, p = 0.56, Spearman’s rank correlation). *Bottom Left:* Scatterplot showing CP5–C4 in the lower alpha band for 40 Hz and Random groups, with a trending positive correlation (rho = 0.27, p = 0.09, Spearman’s rank correlation), suggesting stronger connectivity may relate to higher accuracy. Each dot represents one subject, with red for Random and blue for 40 Hz groups. *Bottom Right:* Scatterplot showing CP5–C4 in the upper alpha band for 40 Hz and Random groups, with no significant correlation (rho = 0.10, p = 0.52, Spearman’s rank correlation). (F) *Left:* Distribution of Spearman’s rho-values for channel pairs with trending or significant (p < 0.1) correlation between functional connectivity (WPLI) and accuracy for lower alpha (green) and upper alpha (grey) for 40 Hz and Light (top) and 40 Hz and Random (bottom). In the 40 Hz and Light subjects, strong correlations between lower alpha WPLI and accuracy were more positive, while strong correlations between upper alpha WPLI and accuracy were more negative (top-left, Chi-squared test for proportions, p = 0.005, Cramér’s V: 0.55). In the 40 Hz and random groups, lower and upper alpha functional connectivity were not significantly different (bottom-left, Chi-squared test for proportions, p = 0.81, Cramér’s V: 0.041) and were both positively correlated with accuracy. *Right:* Topological plot of channel pairs with significant WPLI-accuracy correlations for both lower alpha and upper alpha bands. Positive correlations are shown in orange; negative correlations are shown in purple. The channel pairs are displayed for each comparison between groups: 40 Hz and Light (top) and 40 Hz and Random (bottom). (G) as in E for reaction time. Note that negative correlations with reaction time indicate correlations with faster performance.

Because 40 Hz flicker altered oscillations outside the flicker frequency, we then investigated whether it also altered functional connectivity at frequencies outside 40 Hz. High functional connectivity, indicated by correlated fluctuations in neural activity between different channels, is thought to reflect higher communication or coordination between those regions (Buzsáki, 2009; Fries, 2015). Such coordinated activity is important for cognitive tasks including vigilance (Bogler et al., 2017; Gregoriou et al., 2009; Kamzanova et al., 2020; Oken et al., 2006; Womelsdorf et al., 2006) (Fig. 4A). To assess functional connectivity, we measured the Weighted Phase Lag Index (WPLI) for each pair of EEG channels across each frequency band (Fig. 4B). WPLI measures functional connectivity while minimizing the effects that could be due to volume conduction (or correlations at zero-lag). We used this measure to investigate functional networks engaged in this vigilance task while minimizing spurious covariation due to volume conduction. We identified significant functional connectivity between channel pairs by comparing the measured WPLI to a threshold of 0.089 computed from the shuffled data (p < 0.0001, permutation test) within each frequency band of interest (Fig. 4B). To determine which frequency bands’ functional connectivity was prominent, we first quantified the number of channel pairs with significant functional connectivity in each band (Fig. 4C). Most of the significant functionally connected (FC) channel pairs (374 out of 384 or 97.4%) were within the alpha band in all groups (Fig. 4C). Therefore, we further evaluated how alpha functional connectivity differed between 40 Hz flicker and control groups.

Examining WPLI values as a function of frequency, we noticed that 40 Hz and Light groups differed in their functional connectivity between lower (8–10 Hz) and upper (10–13 Hz) alpha bands (Fig. 4B). The lower alpha band (sometimes called “low alpha”) is associated with basic sensory and baseline attention processes while the upper alpha band (sometimes called “high alpha”) is more sensitive to memory demands (Klimesch, 1999). We found that the peak WPLI frequency within the alpha band was lower in the 40 Hz group (9.35 Hz ± 0.0287 Hz, n = 136 significant FCs) compared to the Light group (9.56 ± 0.0291 Hz, n = 229 FCs; p = 0.000002, ranksum test). Therefore, for further analyses we separated lower and upper alpha. To identify channel pairs with relatively high functional connectivity in each band, we assessed the top quartile of WPLI channel pairs (WPLI>0.12). We found that the 40 Hz group had more channel pairs with high FC in lower alpha than the other groups (Fig. 4D; 40 Hz vs Light: χ² = 34.06, p < 0.0001, Cramér’s V = 0.1853; 40 Hz vs Random: χ² = 82.25, p < 0.0001, Cramér’s V = 0.2879; Chi-squared test; q < 0.1, FDR correction from 2 comparisons). In contrast in upper alpha, the 40 Hz group had fewer channel pairs with high FC than the Light group but more channel pairs than the Random group (Fig. 4D; 40 Hz vs Light: χ² = 48.20, p < 0.0001, Cramér’s V = 0.2204; 40 Hz vs Random: χ²(1) = 188.97, p < 0.0001, Cramér’s V = 0.4365; Chi-squared test; q < 0.1, FDR correction from 2 comparisons). These results show that 40 Hz flicker induced increased FC in more channel pairs within lower alpha than control or sham stimulation.

We then determined whether elevated functional connectivity in lower or upper alpha was correlated with performance in the vigilance task. Because lower alpha is implicated in attention processes, we hypothesized that higher functional connectivity (measured via WPLI) in lower alpha would correlate better with task performance. To see how lower or upper alpha correlated with behavioral performance across subjects and groups, we combined 40 Hz and Random or 40 Hz and Light groups. Because the alpha band FC networks showed different patterns in the 40 Hz, Light, and Random groups, we assessed channel pairs from the 40 Hz and Light groups together and from the 40 Hz and Random groups together to compare correlation patterns between the two group combinations (Fig. 4E). When examining 40 Hz and Light groups, we found lower and upper alpha differed in how functional connectivity was correlated with task accuracy across all channel pairs (Supplementary. Fig. S4B; paired t-test, p = 0.003). More channel pairs had significant or significant-trending positive correlation (p < 0.1) between functional connectivity and accurate performance in lower alpha, while there were more pairs with negative correlations between functional connectivity and accuracy in upper alpha, in line with our hypothesis (Fig. 4F; top-left; Chi-squared test for proportions, p = 0.005, Cramér’s V: 0.55). There were no significant differences between upper and lower alpha in the proportion of channel pairs with strong negative and positive correlations between functional connectivity and reaction time (Fig. 4G; top-left; Chi-squared test for proportions, p = 0.188, Cramér’s V: 0.263). Examining 40 Hz and Random groups, we found both upper and lower alpha bands functional connectivity were positively correlated with accuracy, with no significant differences between the proportion of channel pairs with strong correlations in lower and upper alpha (Fig. 4F, bottom-left; Chi-squared test for proportions, p = 0.8057, Cramér’s V: 0.041). Together, these results show that stronger lower alpha functional connectivity is correlated with more accurate performance in the vigilance task. Thus, the increase in lower alpha functional connectivity in the 40 Hz group is associated with better task performance.

## 4 Discussion

In this study, we found that 40 Hz flicker improved attention in a vigilance task and promoted neurophysiological markers associated with attentional performance. 40 Hz flicker improved accuracy and reaction times in a vigilance task compared to both sham stimulation and constant light, indicating heightened attentional performance. Looking at scalp electrophysiology, we observed expected increases in gamma power, aligning with prior studies on 40 Hz flicker (Attokaren et al., 2023; Herrmann, 2001; Iaccarino et al., 2016). Unexpectedly, we also found that 40 Hz flicker decreased delta power during attention periods and found trends of elevated alpha and beta power. Importantly, decreased delta power was correlated with better behavioral performance. Assessing functional connectivity during attention, 40 Hz flicker increased lower alpha functional connectivity, and this increase was correlated with enhanced task performance, suggesting a link between increased lower alpha coherence and improved vigilance. Previous studies have primarily explored the neural health benefits of *chronic* 40 Hz flicker, especially in the context of neurodegenerative diseases (Bartolini et al., 2025; Chan et al., 2022; He et al., 2021). In contrast, our study offers novel insights into the *acute* effects of 40 Hz flicker on attention and multi-band oscillatory dynamics in healthy adults. By providing evidence of immediate neurophysiological and behavioral effects, we enhance our understanding of how 40 Hz flicker impacts attention-related processes in healthy adults.

This study broadens the application of 40 Hz flicker from previously explored disease applications to attentional enhancement in non-clinical contexts. Furthermore, prior flicker studies examined the effects of flicker on EEG power at the frequency of flicker, while we examined effects across a broader range of frequencies. In line with earlier work, we confirmed 40 Hz flicker modulated power around 40 Hz (39–41 Hz) (Adaikkan et al., 2019; Blanpain et al., 2024; Chan et al., 2022; He et al., 2021; Prichard et al., 2023). Indeed, studies using intracranial recordings have shown that 40 Hz sensory flicker modulates power in local field potentials around 40 Hz with high spatial precision (Blanpain et al., 2024; Chan et al., 2022). Surprisingly, outside the gamma range, 40 Hz flicker decreased delta and increased lower alpha connectivity, modulations that were correlated with improved behavioral performance. Because increased delta power is associated with drowsiness, the decreased delta power we observed during 40 Hz flicker may be due to increased alertness or arousal (Matousek & Petersén, 1983; Oken et al., 2006). Indeed, subjects that received 40 Hz flicker performed faster and more accurately, which is in line with their being more alert in this vigilance task. Alpha oscillations have previously been associated with attention, especially selective attention (Foxe et al., 1998; Foxe & Snyder, 2011; Klimesch, 1999, 2012). Therefore, increased alpha power associated with better performance could indicate that subjects were more successfully suppressing irrelevant information to attend to the color change, the key feature of this task. Interestingly, we found high functional connectivity in lower alpha in the 40 Hz group. In contrast, functional connectivity was high in upper alpha in the Light group. Prior work has shown that alpha frequency decreases with higher attention and memory demands (Klimesch et al., 1993). The observed shift in alpha functional connectivity may reflect a higher attentional demand in the 40 Hz group because the flickering stimuli may require more attentional selection. Alternatively, the high functional connectivity in upper alpha in the Light group may reflect an internally orientated state during which a subject is slower or less likely to respond to an incoming stimulus (Hanslmayr et al., 2011). By documenting changes in both power and functional connectivity across multiple frequency bands, this study provides novel insights into how exogenously induced gamma oscillations affect other oscillatory bands.

Prior work has shown that attention amplifies endogenous gamma-band activity (Jensen et al., 2007). This attention-induced gamma is theorized to enhance sensory processing by increasing the synchronous activity of the neurons responding to that stimulus and thereby making those cells more likely to drive downstream neurons. While periodic stimuli, like the 40 Hz flicker used in this study, generate oscillatory activity in the gamma band, these are not necessarily the same as endogenous oscillations. Indeed, studies have debated whether neural activity in response to rhythmic stimuli is purely sensory evoked or includes modulation of ongoing endogenous rhythms (Zoefel et al., 2018; Duecker et al., 2024, 2021; van Bree et al., 2021). Here, we find elevated power in the Gamma Flicker Response band correlated with improved attentional performance, and we did not try to distinguish sensory driven versus endogenous gamma oscillations. Furthermore, we find that 40 Hz stimulation improves performance in an attention task, suggesting that it may enhance, or at least not interfere with, endogenous gamma activity that underlies attentional processes.

Our results demonstrate the potential for acute 40 Hz flicker to enhance behavioral performance in a vigilance task. This application could be beneficial in jobs where prolonged attention is critical, such as in air traffic control, surgical monitoring, piloting, or long-distance driving. These findings also suggest the potential for flicker-based interventions in non-clinical settings, such as students studying for an exam. However, as our study focused on healthy young adults, additional research is needed to verify these effects in other populations, particularly those with attention deficits.

Our findings have several limitations that pave the way for future studies to further explore 40 Hz flicker’s effects on attention processing. Our study included healthy young adults, and it is unclear if these findings extend to other populations. The acute impacts observed here in healthy adults may differ in other demographic groups, such as clinical populations (e.g., Alzheimer’s, ADHD, epilepsy, etc.) or older adults, who may not respond to 40 Hz flicker in the same way as younger individuals (Bartolini et al., 2025; Chan et al., 2022; Duffy et al., 1984; He et al., 2021; Jabès et al., 2021). Because we measured neural activity using scalp EEG, our findings largely result from cortical activity and provide little insight into the activity of other brain structures. Future studies could investigate the effects of 40 Hz flicker on deep and spatially specific neural structures involved in attention. We identified significantly connected channel pairs in each frequency band, and future work could further dissect how stimulation alters the different spatial patterns of connectivity. In addition, the attention task we used was relatively simple, making it unclear if these effects extend to harder tasks or other types of attention. Furthermore, our study only examined the effects of 40 Hz flicker in a single 1-hour session. Chronic exposure studies could test if 40 Hz flicker yields cumulative attentional benefits, or greater benefits after repeated exposures, further supporting the use of flicker in improving attention performance or in therapeutic interventions for both healthy and clinical populations. Future research could explore whether different types of stimulation—such as audio-only, tactile, invisible spectral (Agger et al., 2022; Hansen et al., 2024), transcranial electrical or magnetic stimulation, or 40 Hz-modulated multimedia—and other stimulation frequencies produce comparable effects.

This study bridges an important gap between therapeutic 40 Hz flicker research in clinical populations and application of 40 Hz flicker in healthy populations. Our findings of immediate faster and more accurate attention performance highlight 40 Hz flicker’s potential as a non-invasive tool for improving attention and suggest applications for attention-demanding contexts. The observed multi-band oscillatory changes extend the current understanding of 40 Hz flicker’s role, offering evidence that exogenous gamma oscillations may serve distinct and complementary functions to endogenously generated gamma rhythms. By identifying acute behavioral benefits and neural changes of 40 Hz flicker, this research supports a wider range of applications and encourages further investigation into the mechanisms and utility of gamma oscillation entrainment across cognitive domains.

## Supplementary Material

Supplementary Material

## Data Availability

Data from this study is available on OpenNeuro https://doi.org/10.18112/openneuro.ds006222.v1.0.0. Code is available on GitHub: https://github.com/singerlabgt/FlickerEEGAttention_HealthyAdults_Manuscript.
