# Supplementary material for: 40 Hz audiovisual stimulation improves sustained attention and related brain oscillations
Source: Imaging Neurosci (Camb). 2026 May 13;4:IMAG.a.1229. doi: 10.1162/IMAG.a.1229 (PMC13175507; doi:10.1162/IMAG.a.1229)
Supplement: Supplementary Material [file IMAG.a.1229_supp.pdf]

SUPPLEMENTARY INFORMATION

40 Hz Audiovisual Stimulation Improves Sustained Attention and Related Brain Oscillations

Authors: Matthew K. Attokaren*[1], Lu Zhang*[1,2], Sindhura Mettupalli[1], Annabelle C. Singer[1]

Contents:
2 Tables
4 Figures

TABLES

Supplementary Table S1. Demographics of Stimulation Subjects per Group

Characteristics	40 Hz	Random	Light	40 Hz vs Random p-value	40 Hz vs Constant p-value
	(n = 21)	(n = 22)	(n = 19)		
Years of Age (mean, (s.d))	20 (1.4)	20 (1.6)	21 (4.0)	0.42	0.29
Female Sex (n (%))	7 (33)	13 (59)	10 (52)	0.13	0.61
Years of Education (mean, (s.d))	14 (1.7)	15 (1.5)	16 (2.3)	0.38	0.12
Race: Caucasian (n (%))	9 (43)	10 (45)	8 (42)	0.99	0.88

Years of Age, Years of Education, and all percentage values are rounded to the nearest integer. Standard deviation values are rounded to the nearest tenth. Student's *t*-tests were used to compare Years of Age and Years of Education. Chi-square tests were used to compare Female Sex, and Race. P-values were not corrected for multiple comparisons.

Supplementary Table S2. Power-Behavior Correlation

		**PSD-Acc**		**PSD-RT**	
**Channel**	**Band**	**rho**	**p-value**	**rho**	**p-value**
Fp1	Delta	-0.495	0.0005	0.4895	0.0008
Cz	Delta	-0.4759	0.0005	0.3359	0.0358
Oz	Delta	-0.457	0.0007	0.3228	0.0358
Fp1	Theta	-0.1421	0.3283	0.1943	0.232
Cz	Theta	-0.0765	0.5874	-0.0077	0.9958
Oz	Theta	-0.2226	0.1284	0.1079	0.539
Fp1	Alpha	0.3517	0.0164	-0.3337	0.0358
Cz	Alpha	0.3428	0.0167	-0.2108	0.2119
Oz	Alpha	0.2528	0.084	-0.1815	0.2513
Fp1	Beta	0.2517	0.084	-0.3136	0.0358
Cz	Beta	0.1199	0.3982	0.0277	0.9958
Oz	Beta	0.1518	0.315	-0.0021	0.9958
Fp1	Slow Gamma	0.1607	0.3068	-0.2081	0.2119
Cz	Slow Gamma	0.0457	0.72	0.0007	0.9958
Oz	Slow Gamma	0.2583	0.084	-0.1591	0.313

Spearman's rank correlation rho-values and p-values for correlations between power and accuracy and between power and reaction time. P-values are FDR-corrected for 15 comparisons (3 channels, 5 frequency bands).

SUPPLEMENTARY FIGURES

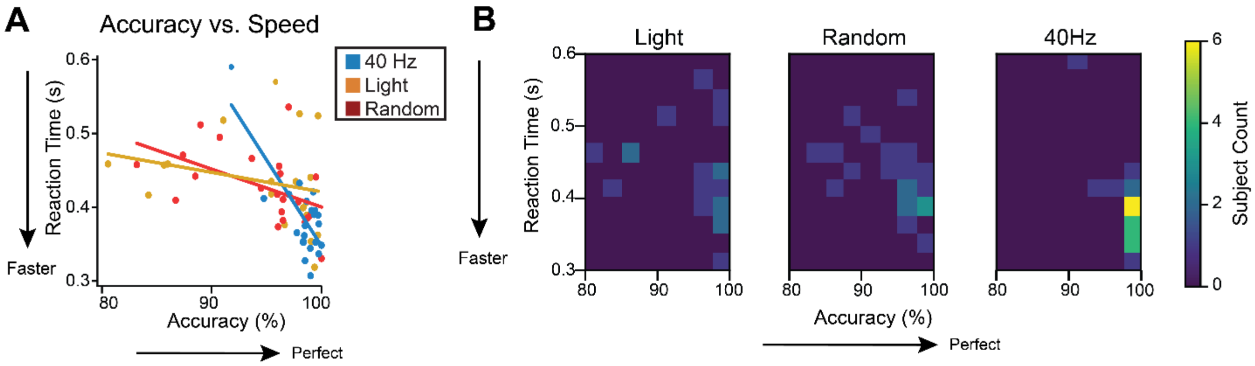

Supplementary Figure 1. Accuracy versus speed relationship during PVT
(A) Average accuracy versus average reaction time per subject for those that underwent 40 Hz flicker (blue; Spearman's rank correlation: Spearman's rho = -0.412, p-value = 0.072, n = 21), Random (red; Spearman's rank correlation: Spearman's rho = -0.569, p-value = 0.006, n = 22), and Constant Light (gold; Spearman's rank correlation: Spearman's rho = -0.412, p-value = 0.079, n = 19). Each dot is one subject. Lines indicate best linear fit (first-degree polynomial fit). Higher accuracy was not correlated with slower reaction times. Instead, there was a trend of a correlation (40 Hz and Light groups) or a significant correlation (Random group) in the opposite direction (faster reaction time was correlated with higher accuracy).
(B) Heatmap of average accuracy versus average reaction time per stimulation group with more yellow colors indicating more subjects in that bin.

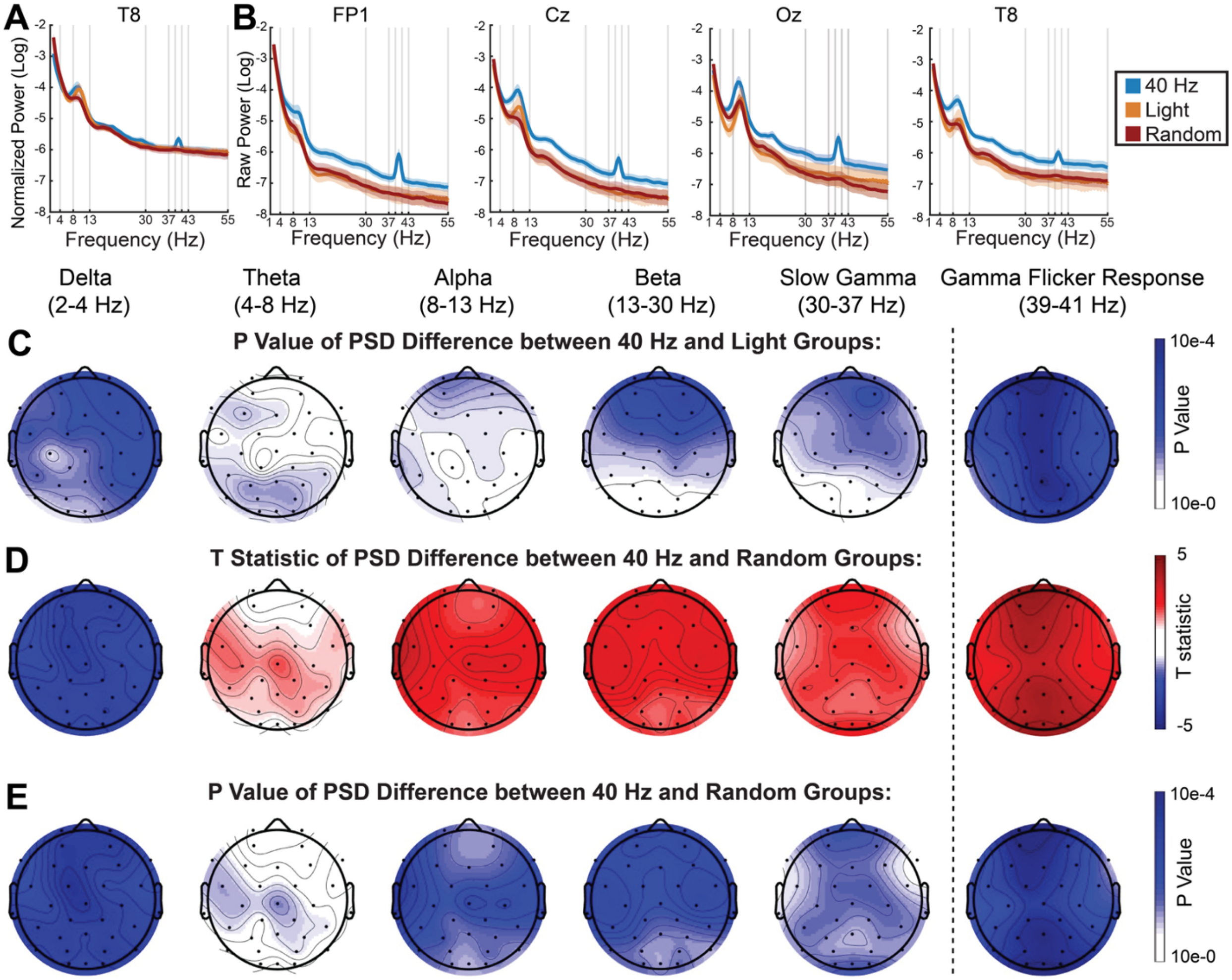

Supplementary Figure 2. 40 Hz flicker decreases delta activity during a vigilance task

(A) Normalized power spectral density (PSD) plot for T8 in the 40 Hz (blue), constant light (orange), and Random (red) groups. Each PSD shows mean +/- SEM

(B) Raw power spectral density PSD plots in the 40 Hz (blue), constant light (orange), and Random (red) groups. Each PSD shows mean +/- SEM

(C) Maps of p-values (not corrected for multiple comparisons) for PSD differences from comparing the PSD of the 40 Hz group and Light groups during the attention period.

(D) PSD difference map showing the *t*-statistic of the difference between the PSD of the 40 Hz group and Random groups during the attention period.

(E) Maps of p-values (not corrected for multiple comparisons) for PSD differences from comparing the PSD of the 40 Hz group and Random groups during the attention period.

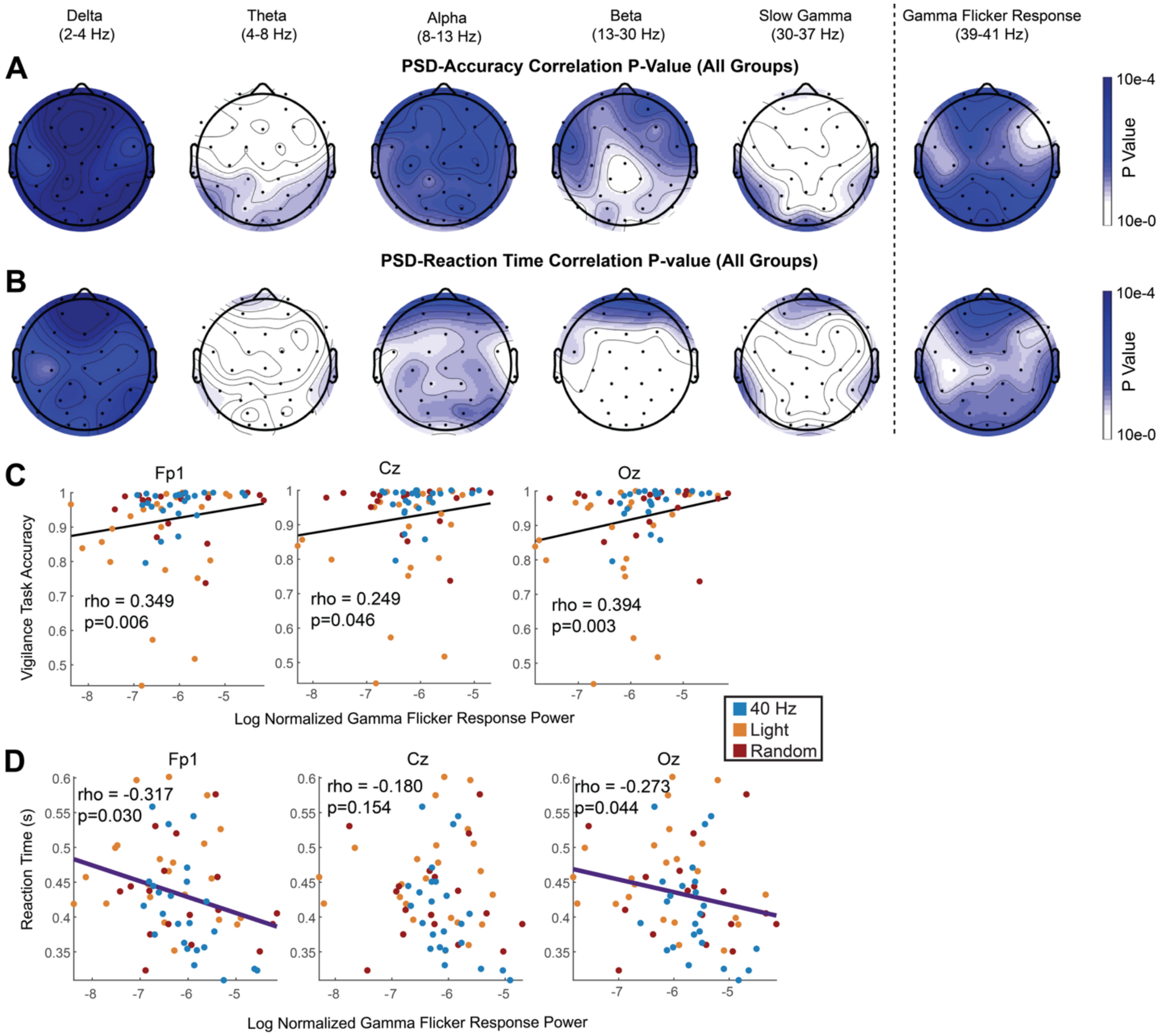

Supplementary Figure 3. Power-behavior correlation maps of p-values.

(A) Maps of p-values (not corrected for multiple comparisons) for power-accuracy correlation across all subjects in all groups. Spearman's rank correlation.

(B) Maps of p-values (not corrected for multiple comparisons) for power-reaction time correlation across all subjects in all groups. Spearman's rank correlation.

(C) Spearman's rank correlation between Gamma Flicker Response band (39-41 Hz) power on Fp1 (left), Cz (center), and Oz (right) and accuracy across all subjects. Each dot represents one participant with dot color indicating stimulation group (blue for 40 Hz, gold for Light, and red for Random).

(D) As in **C** for Gamma Flicker Response band-reaction time correlation.

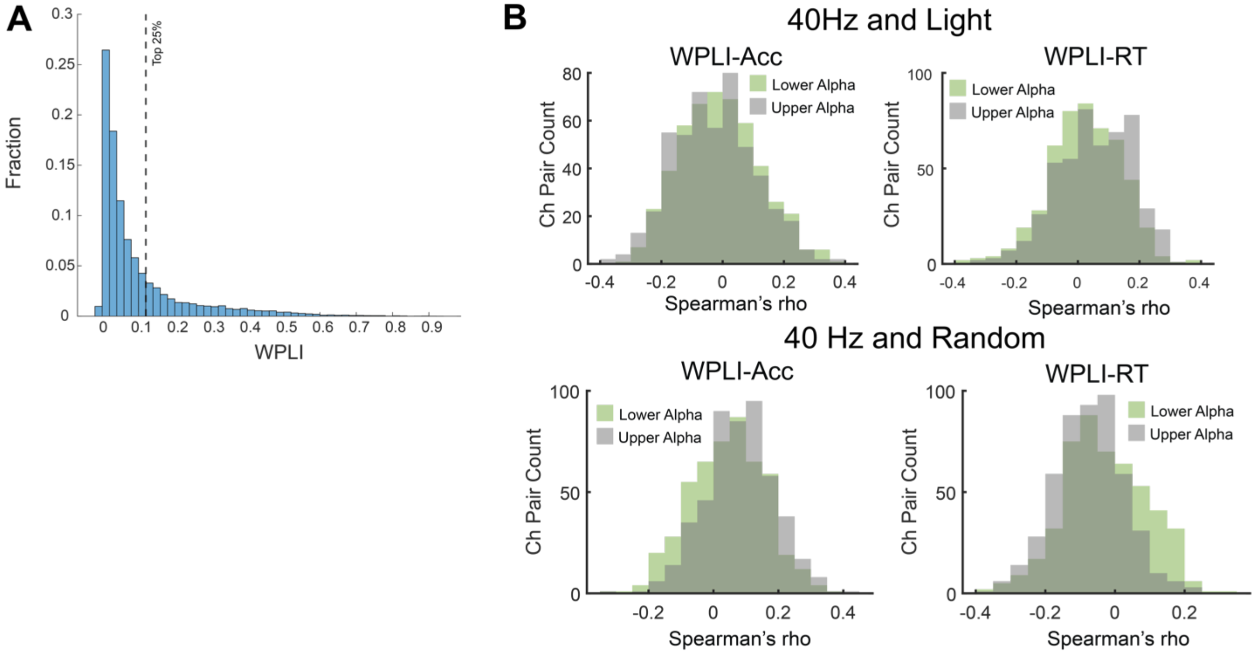

Supplementary Figure 4. WPLI and WPLI-behavior correlation distributions

(A) Distribution of peak WPLI values within the alpha band of all subjects' channel pairs with top quartile (WPLI = 0.12) indicated by dashed line (n = 67 subjects x 496 channel pairs = 33,232 alpha peak WPLI values).

(B) Distribution of WPLI-Behavior Spearman's rank correlation coefficients (rho) across all channel pairs (n=496) comparing lower alpha (green) and upper alpha (grey) frequency bands. *Top left*: Distribution of WPLI-Accuracy Spearman's rho values for the 40 Hz and Light conditions. The distributions of lower alpha and upper alpha were significantly different (paired t-test, p = 0.003). *Top right*: Distribution of WPLI-Reaction Time Spearman's rho values for the 40 Hz and Light conditions. The lower alpha and upper alpha distributions were significantly different (paired *t*-test, $p = 4 \times 10^{-9}$, n=496). *Bottom left*: As in top left for 40 Hz and Random conditions. The lower alpha and upper alpha distributions were significantly different (paired *t*-test, $p = 5 \times 10^{-10}$, n=496). *Bottom right*: As in top right for 40 Hz and Random conditions. The lower alpha and upper alpha distributions were significantly different (paired *t*-test, $p < 10^{-16}$, n=496).
